# Deliberate and Accidental Gas-Phase Alkali Doping of Chalcogenide Semiconductors: Cu(In,Ga)Se_2_

**DOI:** 10.1038/srep43266

**Published:** 2017-02-24

**Authors:** Diego Colombara, Ulrich Berner, Andrea Ciccioli, João C. Malaquias, Tobias Bertram, Alexandre Crossay, Michael Schöneich, Helene J. Meadows, David Regesch, Simona Delsante, Guido Gigli, Nathalie Valle, Jérome Guillot, Brahime El Adib, Patrick Grysan, Phillip J. Dale

**Affiliations:** 1 University of Luxembourg, Physics and Materials Science Research Unit. 41, rue du Brill, L-4422 Belvaux, Luxembourg; 2 Robert Bosch GmbH, Corporate Sector Research and Advance Engineering, Robert Bosch Campus 1, D-71272 Renningen, Germany; 3 Università la Sapienza di Roma, Dipartimento di Chimica, Piazzale Aldo Moro 5 00185 Roma, Italy; 4 Department of Materials Engineering, KU Leuven, Kasteelpark Arenberg 44–bus 2450, B-3001 Leuven, Belgium; 5 NETZSCH-Gerätebau GmbH, Wittelsbacherstraße 42 D-95100 Selb/Bayern, Germany; 6 Università degli Studi di Genova, Dipartimento di Chimica e Chimica Industriale, Via Dodecaneso 31 16146 Genova, Italy; 7 Luxembourg Institute of Science and Technology, 41, rue du Brill, L-4422 Belvaux, Luxembourg

## Abstract

Alkali metal doping is essential to achieve highly efficient energy conversion in Cu(In,Ga)Se_2_ (CIGSe) solar cells. Doping is normally achieved through solid state reactions, but recent observations of gas-phase alkali transport in the kesterite sulfide (Cu_2_ZnSnS_4_) system (re)open the way to a novel gas-phase doping strategy. However, the current understanding of gas-phase alkali transport is very limited. This work (i) shows that CIGSe device efficiency can be improved from 2% to 8% by gas-phase sodium incorporation alone, (ii) identifies the most likely routes for gas-phase alkali transport based on mass spectrometric studies, (iii) provides thermochemical computations to rationalize the observations and (iv) critically discusses the subject literature with the aim to better understand the chemical basis of the phenomenon. These results suggest that accidental alkali metal doping occurs all the time, that a controlled vapor pressure of alkali metal could be applied during growth to dope the semiconductor, and that it may have to be accounted for during the currently used solid state doping routes. It is concluded that alkali gas-phase transport occurs through a plurality of routes and cannot be attributed to one single source.

Control of alkali doping is crucial for a range of technologically relevant chalcogenide materials, from photovoltaics (CdTe, Cu(In,Ga)Se_2_, Cu_2_ZnSn(S,Se)_4_)^1^,^2^,^3^,^4^,^5^ and thermoelectricity (Pb(S,Se,Te))^6^,^7^,^8^,^9^ potentially to superconductivity (KFeSe_2_)^10^,^11^ and quantum computing (Bi_2_Te_3_^12^, MoS_2_ and WSe_2_^13^). In the case of Cu(In,Ga)Se_2_ (CIGSe) solar cell material, the current alkali metal doping procedures are overwhelmingly based on condensed state reactions. Two common approaches are taken. Either by indirect control of the diffusion from a sodium-containing substrate or back contact^14^,^15^,^16^,^17^, or by deliberate doping from the precursor surface through a post deposition treatment (PDT), e.g. by NaF or KF evaporation onto the surface of the absorber to form a tens of nanometer thick layer, followed by annealing^18^,^19^,^20^. Control of the sodium content in the former case is difficult as substrates are never identical^21^, and in the latter case at least one extra step is required to add the alkali metal. The subject has been extensively reviewed by Salomé *et al*.^22^.

CIGSe thin films are always grown in a controlled atmosphere containing a certain pressure of selenium. The semiconductor requires selenium for its formation and to prevent its decomposition, given that the reaction is ruled by a solid/gas-phase equilibrium^23^,^24^. The question arises, are any other gas-phase chemical species involved in the equilibrium? All the main binary compounds of CIGSe have low vapour pressures; however, usually CIGSe contains also a considerable amount of sodium incorporated in the film. Is the vapour pressure of sodium or its likely selenide compounds significant? Does this affect the properties of CIGSe films? Alkali elements or alkali metal compounds may diffuse through a gaseous environment, get absorbed, and react with the semiconductor while it is being synthesized. This would correspond to a *gas-phase alkali doping*.

Remarkably, given the amount of research into alkali doping, the possibility of vapour phase doping has been scarcely considered in the literature, even in the most recent review^22^. This may be due to a lack of phenomenological understanding.

In early 2014 Johnson *et al*.^1^ reported an unidentified vapour phase transport of sodium and potassium under moderate sulfurization conditions. The transport has been observed due to the successful incorporation of the alkali metals in Cu_2_ZnSnS_4_ (CZTS) – a promising candidate absorber layer material for solar cell applications – grown as thin films in the presence of sulfur on alkali-free substrates. This was achieved by simply loading solid NaOH or KOH during the sulfurization of Cu-Zn-Sn precursors at 600 °C in the same reaction chamber. Johnson *et al*. provide a thorough chemical characterization of the resulting CZTS films; however, neither the chemical transport reactions nor the electrical or device properties were investigated, so the technological importance of the incorporation was not assessed.

Nine years earlier than the paper by Johnson *et al*., a striking phenomenon was reported by Wieting *et al*., during batch reactive annealing of CIGSe at Shell Solar^25^. They showed that all CIGSe solar cell power conversion efficiency parameters improved when the CIGSe active layer was annealed facing a soda lime glass substrate compared to when facing another CIGSe layer. They also found an increase of sodium in the CIGSe layer. Since the sodium was not deliberately added, it was hypothesized that it had travelled in the vapour from the glass to the CIGSe. It was proposed that sodium from the glass reacts with H_2_Se to form Na_2_Se, and the latter would then sublime, diffuse to the CIGSe and react. Their proposed reaction is reported below:


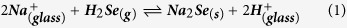



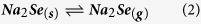


Reaction (1) is equivalent to an ion exchange reaction. Given the decrease of gas molecules, the equilibrium should shift to the left as the temperature increases, but an increase of H_2_Se pressure and the occurrence of (2) should drive it to the right.

Given the reported reaction, one may wonder if only H_2_Se can release sodium from soda lime glass or it is also possible with elemental selenium. Further, the volatility of Na_2_Se would appear to be important, since it would impact on the amount of sodium transported and remaining in the annealing apparatus. If a volatile sodium compound permeates the furnace, then its presence could also accumulate over many annealings. In this way any semiconductor being grown in the furnace would be “accidentally” doped. A further indication that Na_2_Se can supply sodium to CIGSe is given in a recent work by some of the current authors, where a mixture of Na_2_Se and Se was successfully employed for a “deliberate” gas-phase sodium doping of epitaxial CI(G)Se films^26^.

If gas phase alkali metal vapour allows deliberate doping of the chalcogenide during its very formation, with no extra steps, a new strategy for doping is opened, potentially more energy efficient. Such a parallel route may anyway need to be accounted for during condensed state alkali metal doping. Furthermore alkali contamination in fabrication equipment is currently ignored which means that current trends in alkali doping might need careful re-interpretation.

In this work we aim to demonstrate that “accidental” and “deliberate” alkali metal gas-phase doping can significantly affect CIGSe device performance when annealing with elemental selenium. CIGSe is chosen as a case study because for this compound sodium and potassium doping are critical to obtain world record solar cell efficiencies above 20%^2^,^4^. Two common types of annealing chambers are employed, a semi-closed apparatus and a gas-flow system. A first motivation for using two annealing setups is to show the generality of the observations. Secondly, the closed system approaches equilibrium, whilst the flowing system is further from equilibrium, which is more similar to the commonly used physical vapour deposition synthesis tool. Lastly, physical-chemical insights into the proposed equilibria are provided, based on thermodynamic data as well as mass spectrometric studies. The differences observed are related to the thermochamical data for a number of alkali compound sources of doping.

## Experimental Details

### Fabrication of CIGSe with a gas-flow apparatus

The advantage of using a gas-flow apparatus in conjunction with alkali gas-phase transport is that the geometry of the system allows to produce CIGSe library samples with a lateral gradient of doping concentration during the CIGSe growth^26^.

**Figure 1:**
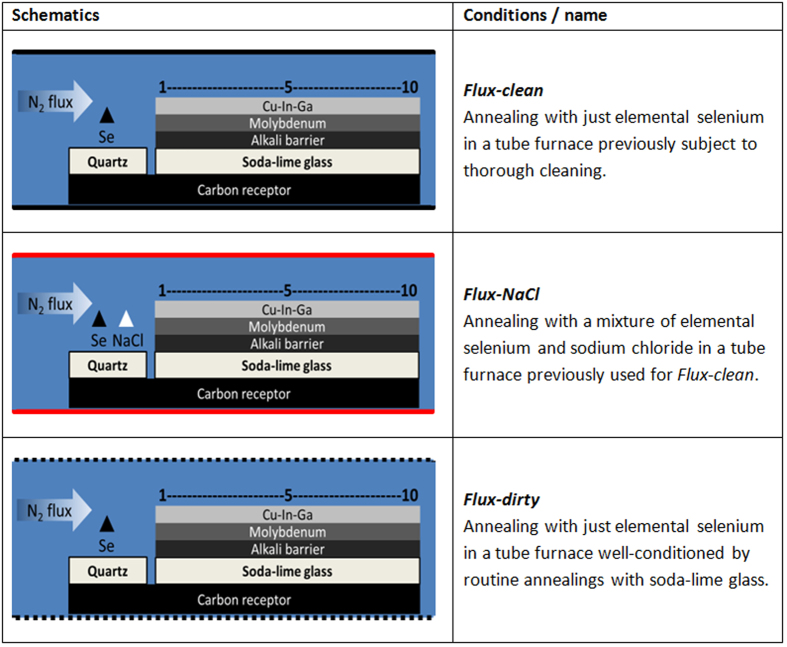
Summary of gas-flow selenization experiments.

For this purpose, a Cu-In-Ga metal precursor film was obtained from solution, as described in ref. 27 on Mo-coated glass substrates with an alkali barrier. The metal precursor was annealed in flowing elemental selenium vapour with a nitrogen carrier flux for 7 minutes. This approach has previously yielded CIGSe solar cells with 13.3% power conversion efficiency, which is among the highest for hydrazine-free solution processed CIGSe^28^. The conditions employed for this set of experiments are summarized in Fig. 1.

Three doping experiments were tested, as described in Fig. 1. The tube furnace employed for *Flux-clean* was mechanically scrubbed of selenium and then heated empty in the presence of flowing selenium at 600 °C twice, in order to produce a blank library sample with no intentional sodium contamination. For the *Flux-NaCl* sample, the selenium powder was replaced with a mixture of NaCl and elemental selenium, to provide a library sample with intentional gas-phase sodium incorporation. The third experiment, *Flux-dirty*, was run again with just elemental selenium, but the tube furnace employed was previously conditioned with a number of routine annealings that involved the use of soda-lime glass (SLG) substrates and various other samples containing Na salts in the precursor. The purpose of this test is to analyse the effects of a typical background sodium contamination.

The metal precursors employed for this set of experiments were approximately 8 cm long and 1.5 cm wide, allowing the fabrication of libraries consisting of a single row of 0.5 cm^2^ individual solar cells (numbering of which is shown in the schematics in Fig. 1). The solar cell finishing procedure employed consists of the sequence CdS/i-ZnO/ZnO:Al/Ni/Al and was performed at ZSW. CdS buffer layers (typically 50 nm thick) were deposited by a conventional chemical bath deposition, the ZnO layers (80 nm + 380 nm) by sputtering and Ni-Al grids (10 nm + 2 μm) by e-beam evaporation. IV characteristics of the devices were recorded with a custom-made solar simulator with illumination intensity set to AM1.5 using a certified silicon solar cell. External quantum efficiency (EQE) measurements of selected devices were performed with a custom-made setup with a chopped double monochromator light beam and a lock-in amplifier, and calibration with Si and InGaAs photodiodes of certified quantum efficiency. Capacitance-voltage curves were recorded by a LCR (Inductance-Capacitance-Resistance) meter, performed under a small AC bias of 30 mV at 100 kHz and room temperature. To minimize the capacitance contribution of deep traps the Mott-Schottky plot was evaluated at small forward biases. Furthermore the phase angle was monitored to verify that the measured value is not dominated by resistive effects, therefore datapoints with a value below 20° were disregarded^29^.

### Fabrication of CIGSe with a semi-closed apparatus

The advantage of using a semi-closed system is that intentional compositional gradients are minimized and the gas-phase transport studied *ex-situ* approaches more closely the thermodynamic equilibrium conditions compared to a gas-flow system.

**Figure 2:**
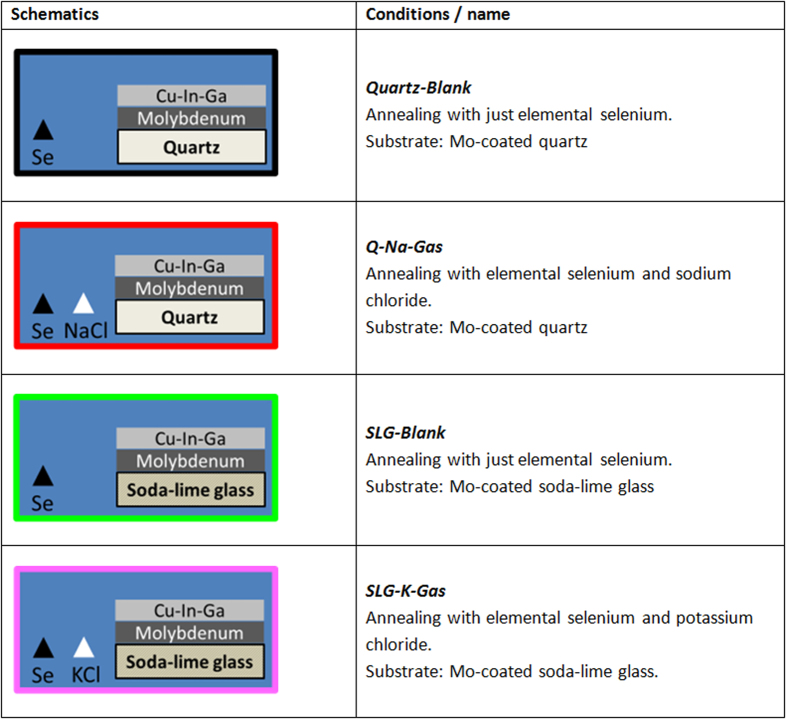
Summary of semi-closed selenization experiments.

For this purpose, Cu-In-Ga metallic precursors were obtained by electrodeposition. Cu layers were electroplated on Mo-coated quartz substrates (sodium free, Ga/(In + Ga) = 0.25) or Mo-coated soda-lime glass substrates (K_2_O content <1.2% wt., Ga/(In + Ga) = 0.40)^30^, followed by In and Ga co-electrodeposition from ionic liquid solutions^31^. Care was taken to employ electrolytes free from intentional sources of sodium (samples *Quartz-Blank* and *Q-Na-Gas* in Fig. 2) or potassium (samples *SLG-Blank* and *SLG-K-Gas* in Fig. 2). A precursor deposited on the quartz substrate was divided into two to form two identical precursors, and similarly the precursor deposited on SLG was cut in half. The precursors were selenized in a graphite box at 550 °C for 30 minutes under conditions listed in Fig. 2.

The first selenization was performed on a Na-free precursor as a blank experiment (*Quartz-Blank*), followed by the selenization in the presence of elemental selenium and NaCl in a 5:1 mole ratio (*Q-Na-Gas*). The precursor on soda-lime glass was also selenized as a standard baseline blank with only elemental selenium (*SLG-Blank*), followed by the selenization in the presence of elemental selenium and KCl in a 5:1 mole ratio (*SLG-K-Gas*). The tubular furnace containing the graphite box employed for the selenization was heated to 800 °C and left under vacuum for 48 h between *Q-Na-Gas* and *SLG-Blank*. The morphology of the absorber layers was characterized by SEM (Hitachi SU-70) using an electron acceleration voltage of 7 kV. A PicoSPM LE atomic force microscope (AFM) operating in acoustic mode was employed to compare the surface roughness of *SLG-Blank* and *SLG-K-Gas*. The compositional depth profiles were measured by SIMS via bombardment with ^133^Cs^+^ ions. The raw ^23^Na, ^39^K and ^35^Cl SIMS signals were normalized against the respective fluxes of ^133^Cs^+^ ions that were recorded simultaneously, in order to provide a basis for sample comparison with minimized matrix effects. Auger electron spectroscopy (AES) depth profiling was carried out on selected samples with a VG Microlab350 operating at 10 kV and 5 nA electron beam and using a 3 kV 1 μA Ar^+^ beam creating a crater of 1 mm^2^. Edge effects or mis-alignements between the argon gun and the electron are avoided by analysing just the central 64 μm^2^ area of film being sputtered.

Photolominescence (PL) analyses were performed with a 514 nm wavelength excitation source (Ar^+^ laser). The PL signal was spectrally resolved in a monochromator (SR-303i-B by Andor) and detected with a Si and an InGaAs detector. The penetration depth of the 514 nm wavelength excitation source employed is expected to be on the order of few hundreds of nm.

### Mass spectrometry and crystallographic analyses

Na_2_Se as purchased from Alfa Aesar (99.8% metals basis) was analysed by Knudsen effusion mass spectrometry (KEMS) in order to measure its vapour pressure as a function of the temperature in close to equilibrium conditions. The powder was handled inside a glovebox as far as possible, due to its high hygroscopicity and sensitivity to oxygen. In the KEMS technique the effusing vapors are analyzed with a mass spectrometer^32^. Details of the apparatus used here can be found in refs 33,34. Briefly, it is based on a single focusing 90° magnetic sector mass spectrometer. The effusing vapor species are ionised by impact with an electron beam whose energy can be changed continuously from appearance of each ion (the minimum energy required to ionize) up to 80 eV. Ion current for a given *m/e* value (single ion monitoring) is measured by an electron multiplier. A movable shutter is interposed between the effusion orifice and entrance to the ionization area to subtract background contributions from the total ion current for a given ion. The background-subtracted ion intensity of the isotope *n* of the species X, 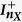, can be converted into the partial pressure of X inside the cell by the equation^32^


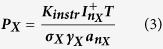


where *T* is the temperature, 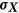 the ionization cross section, 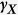 the electron multiplier gain, 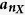 the isotopic abundance, and 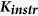 a constant that depends on geometry of the apparatus, cell features and operating conditions. The instrumental constant was measured *ex situ* by repeated vaporization experiments on pure zinc (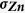 = 5.0 10^−20^ m^2^ 32). A tantalum effusion cell was used with an orifice of 1 mm diameter, inserted in an outer molybdenum crucible. The Knudsen source is heated by irradiation from a spiral-shaped tungsten resistor and the temperature was measured with a Pt/Pt-10%Rh thermocouple inserted in the bottom of the molybdenum container. The mass spectrum of the effusing vapours was studied in the temperature range 450–850 °C. Depending on the temperature, the following ions were observed: Na^+^, NaSe^+^, Na_2_Se^+^, Se_2_^+^. From the analysis of the intensity vs electron energy curves, these ions are assigned to the neutral precursors Na_(g)_, NaSe_(g)_, Na_2_Se_(g)_, Se_2(g)_. In order to estimate the corresponding partial pressures, the cross section of Na was taken from ref. 35 (4.88 10^−20^ m^2^) and those of the molecular species were estimated by multiplying the sum of atomic 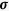 for the empirical coefficient 0.75. To this end, the value 5.90 10^−20^ m^2^ 32 was used for 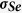. Less extensive measurements were carried out on the Se:NaCl 5:1 mole ratio mixture used during the *Q-Na-Gas* experiment and Se:KF:KCl 1:1:1 in the temperature range 300–700 °C. In this case, alumina and graphite Knudsen cells were preferred, respectively. As a rule, the KEMS measurements were carried out with an electron energy corresponding to the maximum of the ionization efficiency curve of the selected ion.

Additionally, a S:KOH 5:1 mole ratio solid mixture was loaded in a Pt beaker without lid and analysed with a Netzsch STA 409 thermogravimetry (TG) instrument coupled with a quadrupole mass spectrometer (QMS) either through an Aëolos or through a Skimmer system. The thermobalance was operated with a heating rate of 10 °C/min under a 70 ml/min Ar flux. Only the analysis using the Skimmer system proved useful in detecting the target molecules, most probably because of condensation occurring on the walls of the Aëolos setup. X-ray diffraction (XRD) analysis of the solid residuals of the TG-MS run was performed to identify the crystalline phases produced during the themal treatment. For this purpose, a Philips X’Pert MPD was employed in θ−2θ configuration, equipped with a copper target, excited to 40 kV and 30 mA, and a solid state detector (2θ resolution: 0.02° and acquisition time: 7 seconds per step). The crystalline phases have been identified using POWDER CELL software and the Pearson Database for the structural data.

## Results and Discussion

This section consists of five parts. In the first part, the significant effect of accidental and deliberate sodium gas phase doping during CIGSe growth on device opto-electronic properties including efficiency is demonstrated. In the second part, the effect of alkali gas phase doping on the absorber layer microstructure and alkali concentration profiles is reported. In the third part, the mechanism of alkali gas phase generation and transport is discussed based on the mass spectrometric data and on the thermochemical computations. The fourth part provides preliminary evidence of gas-phase desorption of sodium from CIGSe films. In the final part, the consequences of alkali gas phase transport are discussed on a broader context.

### Relevance of deliberate and accidental gas-phase sodium doping to solar cell properties

**Figure 3:**
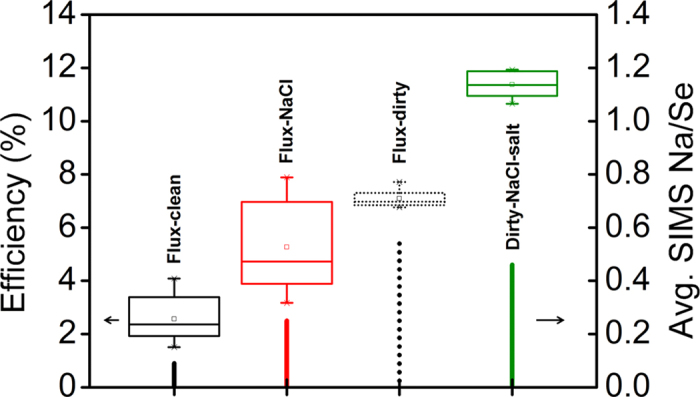
Solar cell device efficiencies of the three CIGSe library samples annealed under nitrogen flux showing the corresponding average, median and spread of values (left ordinate). The vertical lines indicate the average sodium SIMS signal normalized by the Se signal of the first device (right ordinate). The green extra data corresponds to three samples obtained under the same conditions as *Flux-dirty* except that additional NaCl salt was dissolved in the precursor ink (*Dirty-NaCl-salt*, re-plotted from^28^).

Accidental and deliberate gas-phase sodium doping is demonstrated by the results of the gas-flow experiments (section on fabrication of CIGSe with a gas-flow apparatus). The effect of sodium doping is assessed by comparing the solar cell device efficiencies of the three CIGSe library samples. Figure 3 shows the efficiency data spread for each of the samples, along with the average sodium concentration reported as Na/Se SIMS signal.

The *Flux-clean* sample provides the lowest average efficiency (<3%), followed by *Flux-NaCl* (5%) and *Flux-dirty* (7%). The presence of NaCl next to the selenium source during absorber annealing in a clean oven improved the device efficiency by 2% absolute, however “something” in a *dirty* oven improved the efficiency by 4% absolute. The “something” is assumed to be some source of vapour phase sodium since the *Flux-dirty* sample contains nearly six times more Na than the *Flux-clean* sample. The *Flux-NaCl* sample contains 2.5 times the sodium than the *Flux-clean* sample, which also suggests that NaCl acts as a source of gas-phase sodium. The increased efficiency is consistent with the incorporation of sodium, as it reflects its known effect on the CIGSe optoelectronic properties, but importantly the incorporation occurs solely via the gas-phase.

The average device efficiencies are relatively low, but the subsequent observations are valid nevertheless. In order to demonstrate that they are not limited by the CIG precursor or the annealing routine itself a fourth sample is added to the graph corresponding to devices obtained under exactly the same conditions as the *Flux-dirty* library except for the presence of additional NaCl intentionally dissolved in the CIG precursor (*Dirty-NaCl-salt*). Under these annealing conditions an average efficiency of 11% is achieved, with longer annealing times leading to a hydrazine-free record of 13.3% power conversion efficiency^28^. It is interesting to observe that despite the additional sodium in the precursor, the absorber layer itself does not contain the highest amount of sodium. This presumably indicates that sodium can also be lost from an absorber layer during growth. This possibility is investigated in section *gas-phase alkali desorption*.

Further insight can be gained by considering also the spread of values shown in Fig. 3 (full cell parameters are provided as supporting information). The spread of efficiency values is narrowest for the *Flux-dirty* devices indicating the good uniformity of the CIG precursor and of the annealing environment. The *Flux-clean* devices show a slightly wider dispersion of efficiencies but the *Flux-NaCl* devices show the widest range spanning from the *clean* to the *dirty* devices.

**Figure 4:**
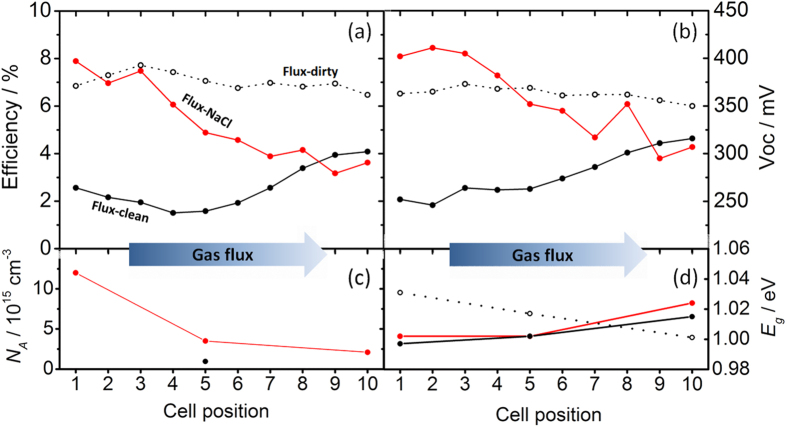
Spatial distribution of efficiency (**a**), open circuit voltage (**b**) and EQE-extracted band gap values (**d**) of the three CIGSe library samples with respect to the geometry of the gas-flow apparatus employed for the selenization. Cell 1 is nearest to the sources and cell 10 furthest away. The net hole doping density of cells 1, 5 and 10 of sample *Flux-NaCl* are shown in (**c**), along with cell 5 of the *Flux-clean* sample.

Figure 4a,b displays the efficiency and open circuit voltage (V_oc_) of each individual device of the three CIGSe library samples. The *Flux-NaCl* solar cell library shows a clear trend of decreasing efficiency moving away from the sodium source, which is driven by a decreasing open circuit voltage. The trend of V_OC_ could be due to a spatially varying band gap (*E*_*g*_) or net hole doping density (*N*_*A*_). However, the subtle variation of band gap cannot explain the observed trend as it actually goes in direction opposite to the V_oc_ (Fig. 4d) and is discussed later. Instead, Fig. 4c shows that *N*_*A*_ increases for the cells closest to the NaCl source. In devices that are not limited by the diffusion length, an increase of *N*_*A*_ has the effect of reducing Shockley-Read-Hall bulk recombination in the material^36^, thus increasing V_OC_ according to: 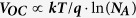
37.

From these doping density results, it is inferred that the V_OC_ and efficiency trend arise from a gas-phase sodium species originating from the Se + NaCl mixture and transported by the N_2_ flux through the tube furnace. As the sodium-bearing species condense on or get absorbed by the growing CIGSe film, locations of the absorber closest to the source (cell 1) will experience higher concentrations of the species during growth compared to locations further away (cell 10). Recently, some of the authors also reported an increase in *N*_*A*_ when an epitaxial CISe film was exposed to Na_2_Se vapour^26^. As a comparison, no such voltage gradient is observed for sample *Flux-dirty* library, where it appears that the distribution of gas-phase sodium-bearing species is most uniform. Interestingly, the sample *Flux-clean* shows an upswing of efficiency and V_OC_ near cell 10. This is attributed to the condensed selenium residue remaining at the end of the tube after the cleaning procedure described in the experimental part. Mass spectrometric analysis of the residue revealed a concentration of sodium of 84 mg/kg, while as-purchased, the selenium contained only 4 mg/kg. This sodium-contaminated selenium residue was nearest cell 10, and thus acted as a sodium source during annealing, resulting in the observed upswing of cell parameters towards cell 10 of sample *Flux-clean*. This means that, despite the efforts, a sodium-free environment was not achieved, although the central cells of sample *Flux-clean* probably experienced the least concentration of gas-phase sodium species, consistent with the low measured *N*_*A*_. This is yet another evidence for the existence of the gas-phase nature of sodium transport. The result highlights the difficulty to achieve sodium-free environments, which implies that gas-phase sodium incorporation of CIGSe is probably unavoidable and needs to be taken into account when extrinsic doping is the subject of research on chalcogenide materials in general. The *Flux-dirty* library shows that the background sodium contamination of a furnace coming from routine experiments can be substantial and acts as a parallel doping source in virtually all literature studies (Fig. 4). Its effect is largely beneficial, but it appears to be insufficient with respect to doping the CIGSe by NaCl supply directly in the precursor ink (Fig. 3). It should be stressed at this stage that the final quantity of Na in the film does not appear to relate to the efficiency. Arguably, a critical Na concentration appears necessary during the growth in order to achieve good optoelectronic properties^38^, but the final concentration of Na will also depend on the film microstructure, as discussed in the next section.

**Figure 5:**
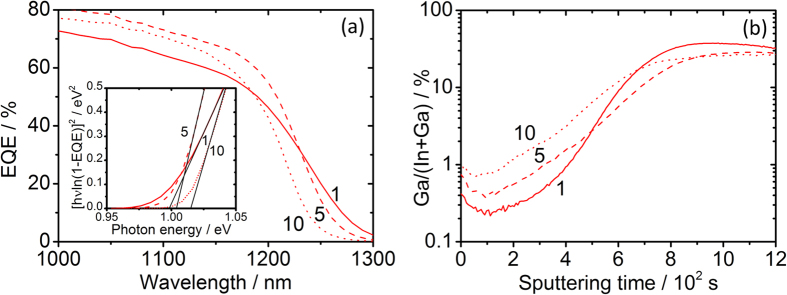
(**a**) EQE measurements of cells 1, 5 and 10 of sample *Flux-NaCl* showing an increasing CIGSe surface band gap (inset) due to (**b**) increasing gallium concentration away from the sodium source, as revealed by the Ga/(In + Ga) SIMS signal ratio.

The monotonic decrease of open circuit voltage shown by sample *Flux-NaCl* moving away from the sodium source actually coexists with a slight opposite trend of surface band gap, as estimated from quantum efficiency measurements. The slight increase of bandgap moving away from the sodium source is consistent with the reported hindering effect of sodium on In/Ga interdiffusion in polycrystalline CIGSe^18^, which is confirmed by SIMS measurements. As such, it further supports the existence of the gas-phase sodium transport. EQE and SIMS measurements of cells 1, 5 and 10 of sample *Flux-NaCl* are shown in Fig. 5.

Summarising this part, it was shown that CIGSe device efficiency is improved from 2% up to nearly 8% by only gas phase sodium species absorption and doping during absorber annealing. Significantly, this could be achieved deliberately by annealing the precursor with a non-classical sodium source, NaCl, sitting next to the selenium source. Equally significantly, this could also be achieved by annealing the precursor in an environment where many previous samples had been annealed. The deliberate doping was achieved in a flowing apparatus and thus the rate of doping and the total amout of doping is very specific to the methodology. The following section shows the results of the experiments with the semi-closed apparatus. In this case, the aim is to better study the mechanism of transport and incorporation in conditions that are closer to equilibrium. To this end focus will be given to the compositional and microstructural effects of gas-phase sodium and potassium doping of CIGSe.

### Gas-phase alkali incorporation: microstructure and composition

In this part the focus is on singling out the effects of deliberate alkali gas phase doping on the CIGSe absorber layers themselves. To avoid accidental alkali metal doping all the selenizations were conducted in a cleaned semi-closed environment in contrast to the last section. The effect of alkali metal gas phase absorbtion on the films surface microstructure has been assessed using optical, scanning electron (SEM) and atomic force (AFM) microscopies.

**Figure 6:**
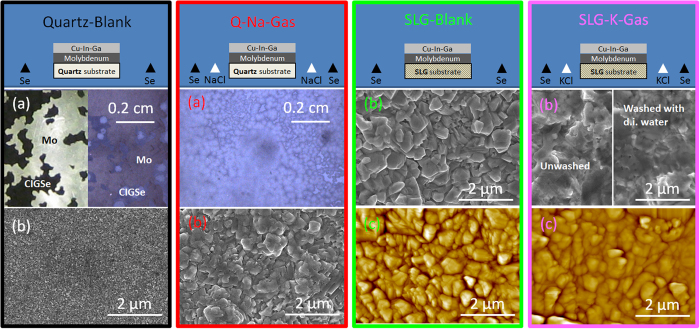
Optical (**a**), SEM (**b**) and AFM (**c**) micrographs of the CIGSe films obtained with the semi-closed apparatus.

The optical microscope images in Fig. 6 show that the *Quartz-Blank* sample displays a very poor adhesion to the underlying Mo film compared to *Q-Na-Gas*. The poor adhesion in the *Quartz-Blank* case is most likely related to compressive stress in the CIGSe layer upon cooling since quartz has a low thermal expansion coefficient compared to Cu(In,Ga)Se_**2**_^39^. It is interesting to note that sodium appears to alleviate the stress, but the effect is not investigated further. The SEM surface images are shown in Fig. 6b. A remarkable enlarging effect of the sodium doping on the surface microstructure is apparent. Sample *Quartz-Blank* displays surface grain sizes smaller than 0.1 μm, while nearly 0.4 μm grains are obtained when sodium doping is achieved via the gas phase, and up to 1 μm in the case of *SLG-Blank*. Besides CIGSe grain-growth enhancement by sodium observed after solid state PDT^16^,^40^,^41^, similar results are reported for CdTe^42^ as well as Cu_2_ZnSn(S,Se)_4_^19^,^20^, where it was proposed to occur by assistance of liquid phase Na-Se compounds. The effect seems similar to the CZTS case, as shown by Johnson *et al*. for gas-phase sodium doping^1^, while Wieting did not show microstructural analyses^25^.

The effect of potassium gas phase inclusion on surface microstructure is also notable, *SLG-Blank* shows an even more pronounced sintering effect, i.e. the sharpness of the grain edges appears to be smoothed and intergrain troughs filled. Atomic force microscopy (AFM) analysis reveals that *SLG-K-Gas* displays half the surface roughness of *SLG-Blank* (0.1 μm versus 0.2 μm). Potassium PDT via KF evaporation on the CIGSe films and selenization has been reported to alter the film surface, leading to improved CIGSe/CdS junction quality and solar cell efficiency^2^. However, the surface morphology obtained here looks very different from that of a typical CIGSe film subject to KF PDT^43^. The CIGSe surface of *SLG-K-Gas* seems free from 100 nm-sized cubic crytals typically observed with the self-assembled alkali-template method shown by Reinhard *et al*., and unlike in their case, the morphology remains unchanged after washing the film in deionized water (Fig. 6b)^44^. This difference may imply that the gas-phase K transport in the present work occurs at a lower rate than a conventional evaporation/condensation of the alkali salt on the CIGSe surface. A lower rate of KCl transport to the surface of the growing CIGSe means that KCl diffusion into the film is favoured over KCl crystallization on the CIGSe surface. This could explain the different surface microstructure and the absence of any effect upon rinsing, compared to Reinhard *et al*.^44^.

**Figure 7:**
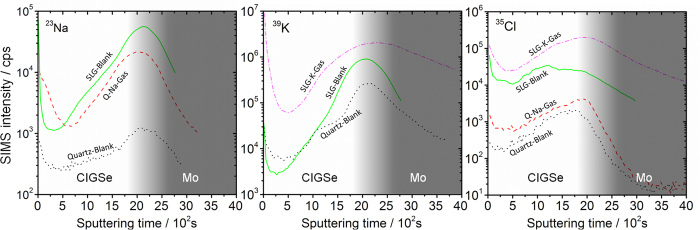
^23^Na (top), ^39^K (centre) and ^35^Cl (bottom) SIMS depth profiles of samples *Quartz-Blank, Q-Na-Gas, SLG-Blank* and *SLG-K-Gas*. The shaded area is a guide for the location of the CIGSe/Mo interface.

The sodium and potassium SIMS depth profiles through the CIGSe films are shown in Fig. 7a,b on two separate semilogarithmic plots. In qualitative terms, the curves show Na and K enrichment at the CIGSe surface, depletion in the CIGSe bulk and increased concentration at the CIGSe/Mo interface. These observations are in line with literature reports^2^,^41^,^45^.

At the current level of understanding it is not possible to ascertain whether the presence of sodium and potassium detected in the bulk of the absorbers is mainly related to the grain boundaries or it is truly incorporated within the grains^46^,^47^. More advanced techniques such as atom-probe tomography are required to obtain detailed topographical resolutions^48^. However, higher affinity of Na for surfaces and grain boundaries has generally been reported^49^,^50^. Therefore, the higher SIMS signals detected at the front and back interfaces hint that a large part of Na and K is associated to grain boundaries, because the density of grain boundary is higher at the interfaces and lower at the centre of the films. Since also for the gas-phase cases Na and K concentrations are high at the CIGSe/Mo interface, alkali diffusion through the developing CIGSe film seems facile, as supported by a recent theoretical work for Na^51^. It is known that diffusion through grain boundaries is faster than in the grain interior^49^, but Na incorporation is likely to occur also within the grains, as confirmed by works on epitaxial CISe films published by some of the authors^26^,^52^.

Some key differences between the samples are observed. In sample *Q-Na-Gas*, where the sodium is supplied from the front surface via the gas phase, the surface sodium concentration is higher compared to *SLG-Blank*, where diffusion comes from the substrate. Furthermore, the sodium level is always higher compared to the *Quartz-Blank* case throughout the whole CIGSe film.

**Figure 8:**
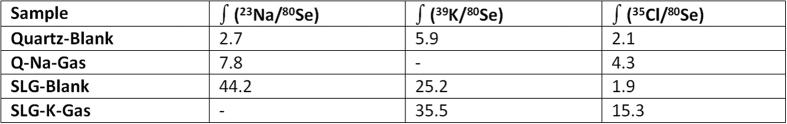
Integrated ^23^Na/^80^Se, ^39^K/^80^Se and ^35^Cl/^80^Se SIMS signal ratios from the samples grown under semi-closed conditions from the layer surface to the sputtering time corresponding to 50% of the maximum ^98^Mo signal.

To roughly estimate the total amount of alkali in each of the films, the corresponding integrations for the ^23^Na and ^39^K SIMS signals are reported in Fig. 8, normalized against the corresponding ^80^Se signals, which are assumed not to deviate substantially from stoichiometry. It should be stressed that the data in Fig. 8 is semi-quantitative. The integrated ^23^Na/^80^Se SIMS ratio of *Q-Na-Gas* is approximately one sixth that of *SLG-Blank*, but it is roughly three times higher compared to *Quartz-Blank*.

The integration of SIMS signals reveals that potassium incorporation in *SLG-K-Gas* is 40% higher than for *SLG-Blank* and six times higher than *Quartz-Blank*. Similarly to the sodium profiles, potassium localizes mostly at the CIGSe/Mo interface, but it is much more evenly distributed in *SLG-K-Gas*. This indicates that K diffuses from both the gas and back interfaces, whereas in *SLG-Blank* it occurs mostly from the back of the film.

Surprisingly the background ^23^Na and ^39^K signals of the blank layer on quartz substrate are not negligible. Actually, these values were achieved after several attempts to reduce the background contamination of alkali metal. This aspect is discussed in the supporting information, but briefly, cleaning was done by repeatedly rinsing the furnace assembly with deionized water and running it at 800 °C for several hours. Failure to do so, i.e. by simply annealing a blank in sequence after multiple runs where SLG substrates were used, resulted in significant increase of the sodium signal. This is a further confirmation of the results shown in Fig. 3 for sample *Flux-dirty* and restate the importance of background alkali contamination for the correct interpretation of a number of research studies on alkali metal doping, where lack of sound trends are lamented^38^,^53^. Especially for the case of sodium, its quasi ubiquitous presence can be a source of unexpected contamination, and therefore of confusion for the interpretation of experimental results^54^.

Of course, in light of a potential development of gas-phase doping strategy in CIGSe films, one legitimate concern is that the halogen could also diffuse into the chalcogenide lattice and alter its optoelectronic properties, namely decrease its *p*-type conductivity due to formation of compensating donors. This may happen, despite Cl substituting Se should be thermodynamically less favourable compared to the Cu replacement by Na and K, due to the different electronic outer shell configuration of Se and Cl.

In order to assess if Cl incorporation occurs in parallel to the alkali metals, the SIMS signal of ^35^Cl has also been measured for all samples and is shown in Fig. 7 and integrated as ^35^Cl/^80^Se ratio in Fig. 8. Incorporation of some Cl does occur during the NaCl gas treatment, as the integrated signal doubles with respect to the blank sample. However the corresponding increase of Na for these samples is roughly three times. Additional Auger electron spectroscopy (AES) depth profiling has been carried out and no Cl signal was detected for the NaCl case, except at the very surface (1–2 at.%, dropped below detection limit at a depth of 50 nm), implying that its amount within the film is below the detection limit (<0.5 at.%).

The situation, is different for the case of KCl. Sample *SLG-Blank* shows a similar ^35^Cl/^80^Se signal ratio compared to *Quartz-Blank* revealing that incorporation of Cl impurities from the SLG substrate into CIGSe is negligible. However, the ^35^Cl/^80^Se ratio increases by one order of magnitude, in the case of *SLG-K-Gas*. A possible explanation for this phenomenon is suggested by thermochemical data and is discussed in section *gas-phase alkali generation and transport: how does it happen?*

Computational studies suggest that *p* to *n*-type polarity inversion under optimum conditions for halogen incorporation in CISe is only achieved at maximal Se-poor conditions, i.e. when CISe coexists with InSe and elemental Cu. In such a case, halogen doping comes mostly from the intrinsic In_Cu_ rather than Cl_Se_ defects^55^. It is to be noted that NaCl was also employed as a deliberate dopant leading to the 13.3% record efficiency CIGSe^28^ (Fig. 3). At this stage it remains to be ascertain if Cl impurity incorporation is to be blamed as the barrier to even higher solar cell efficiencies. These aspects are important in light of device fabrication at industrial level, and should be investigated in a dedicated work.

**Figure 9:**
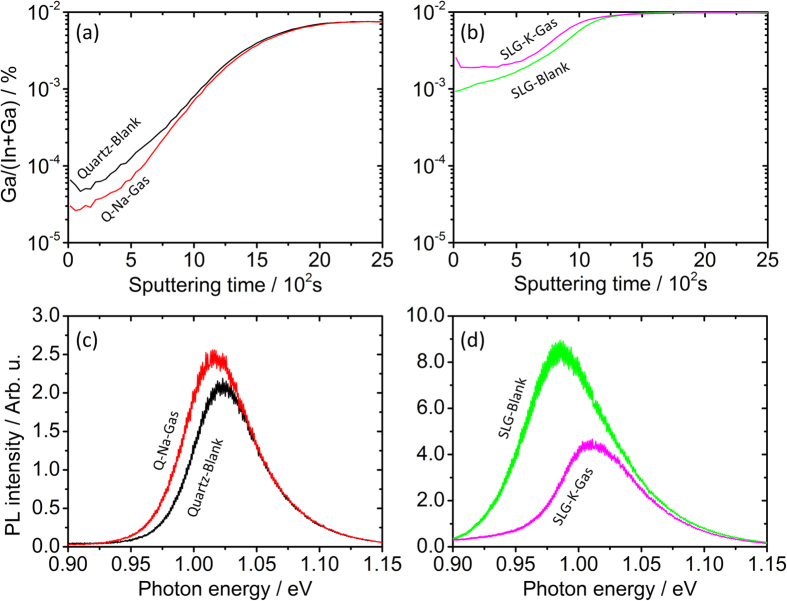
Depth profiles of Ga/(In + Ga) SIMS signal ratios (**a**,**b**) and PL spectra (**c**,**d**) of samples *Quartz-Blank* and *Q-Na-Gas* (**a**,**c**) as well as *SLG-Blank* and *SLG-K-Gas* (**b**,**d**).

The known hindering effect of Na on In-Ga interdiffusion in CIGSe observed with the flux experiment is also confirmed with the semi-closed setup. Figure 9a shows the depth profile of the Ga/(In + Ga) SIMS signal ratios of samples *Quartz-Blank* and *Q-Na-Gas*. A clear Ga depletion is noticed in the film upon Na incorporation. This is also confirmed by a slight decrease of the CIGSe bandgap assessed by photoluminescence measurements^56^ (Fig. 9c).

Interestingly, the opposite behavior is observed upon KCl treatment, as revealed by the corresponding SIMS and PL analyses shown in Fig. 9b,d.

### Gas-phase alkali generation and transport: how does it happen?

The preceding two parts have shown that accidental or deliberate alkali metal vapor phase doping during synthesis is sufficient to significantly alter the properties of the final CIGSe absorber layer. In order to do this, alkali gas species must form, be transported and get absorbed at temperatures compatible with routine CIGSe processing. On the one hand, there are good chances that accidental incorporation of alkali occurs in all routine setups. On the other, the phenomenon has the potential of being exploited in research and/or in industry. It can be anticipated that the mechanism of gas-phase transport must play a key role in determining the extent and location of the alkali dopants in the chalcogenide film, both microscopically and at large scale. Therefore, in this part a chemical explanation of the alkali gas-phase transport is attempted.

Five general possibilities for the reaction and transport of alkali gas-phase compounds are identified and described below (where ***Ak*** is the alkali, ***Ch*** is the chalcogen and ***An*** is the generic anion).

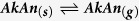
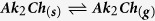
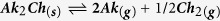
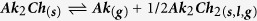
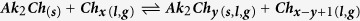

**Figure 10:**
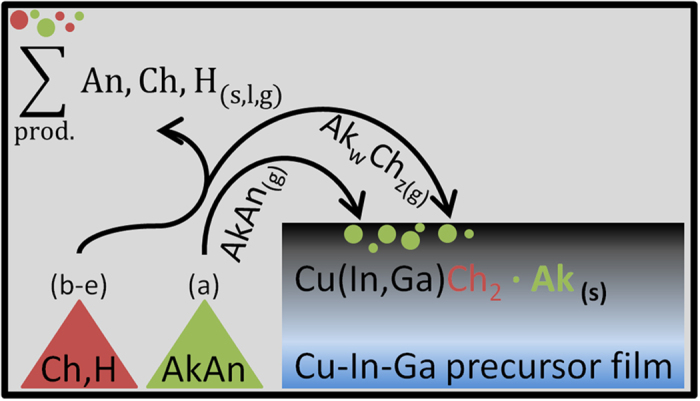
Schematics of gas-phase alkali compound generation, transport and incorporation into the chalcogenide semiconductor (CIGSe here) without (**a**) and with (**b**–**e**) assistance of elemental chalcogen and/or hydrogen. The left top corner indicates the contamination of the annealing chamber by the residual products.

Conceptually, these five possibilities can be reduced to two scenarios, as shown in the schematics of Fig. 10.

Route (a) is the simplest possible. Here a congruent evaporation of the alkali compound occurs, followed by transport via the gas-phase, condensation and incorporation into the film. NaCl and KCl are the alkali compounds used in this work. The alkali chlorides are not known for their high volatility, but their reported vapor pressures at 550 °C, a typical growth temperature for chalcogenide semiconductors, are in the order of 10^−3^ mbar and are therefore non negligible (Fig. 10)^57^. The alkali fluorides are more commonly used in the literature, and their vapor pressure is lower than the chlorides^22^.

Route (a) could well explain the KCl case in this work. Indeed, the SIMS data in Figs 7 and 8 show that K and Cl increase similarly, hinting to a large contribution of direct KCl evaporation to the alkali transport. However, the same conclusion cannot be drawn for the NaCl case. The large disparity between Na and Cl incorporations implies that simple NaCl evaporation is not the main (or only) transport route if the CIGSe film is to remain charge neutral. It follows that other gas species need to be taking part in the gas-phase transport in this case.

Routes (b) to (e) are based on the formation and subsequent outcome of Ak_2_Ch. Ak_2_Ch can form by reaction between the alkali compound and the elemental chalcogen in the presence or absence of gaseous hydrogen, as per reactions (4).


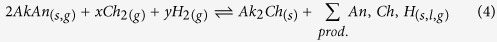


Reactions (4) can occur in the condensed state, as well as in the gas-phase. Figure 12 gives a list of possible reactions, (5–11), along with their variation of standard Gibbs free energies calculated at 550 °C based on literature data^58^,^59^. Here, only the formation of dialkali monochalcogenides is considered, because Gibbs free energies of formation of the polychalcogenides are not available.

The Ak_2_Ch formed can then contribute to alkali transport by congruent evaporation (b), dissociation and release of atomic alkali (c-d) or formation of alkali polychalcogenide species by reaction with chalcogen (e). These possibilities are discussed next, based on literature data and new mass spectrometric measurements.

**Figure 11:**
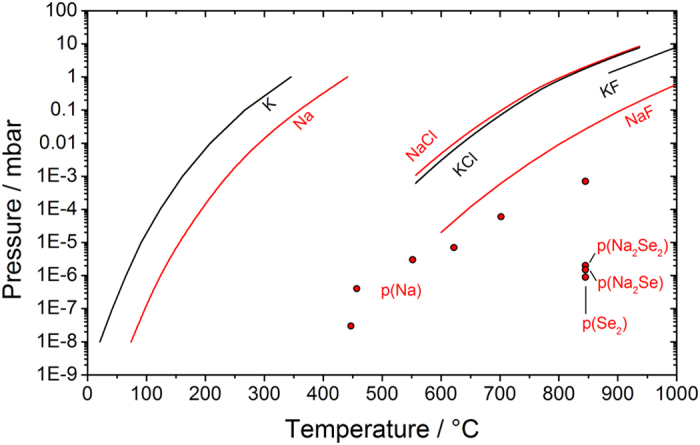
Equilibrium vapor pressures of elemental Na and K^60^, NaCl and KCl^57^ as well as NaF^75^ and KF^76^ on a semilogarithmic scale as a function of the temperature. The data corresponding to the Knudsen effusion mass spectrometric analysis of pure Na_2_Se (Alpha Aesar) is plotted on the same graph (scattered points).

Figure 11 shows the vapor pressures of elemental Na and K^60^, as well as the chlorides^57^ and fluorides, along with new data corresponding to pressures recorded by Knudsen effusion mass spectrometry (KEMS) in this work for undissociated gaseous Na_2_Se, atomic Na, Na_2_Se_2_ and Se_2_ from Na_2_Se decomposition.

It is clear that Na_2_Se decomposes incongruently mostly by losing atomic Na. A build-up pressure of Na below 10^−5^ mbar is recorded at around 600 °C for Na_2_Se solid at early stages of decomposition. The KEMS data on pure Na_2_Se points to an extremely low pressure of undissociated Na_2_Se under equilibrium effusion conditions. (it is only detected at 845 °C with a pressure of ca. 10^−6^ mbar). It follows that route (b), advocated by Wieting *et al*.^25^, seems unlikely. Furthermore, the very small pressure of Se_2_ recorded (<10^−6^ mbar at 845 °C) suggests that also route (c) should be discarded. In fact, if (c) was the main dissociation route, a selenium pressure would be expected to follow closely the Na evolution starting from 450 °C (all forms of selenium are gaseous above 685 °C, and selenium vapor pressure exceeds 10 mbar above 344 °C^59^). On the other hand, the small pressure recorded for Na_2_Se_2_ (ca. 10^−6^ mbar at 845 °C) is an indication that route (d) is possible. Route (d) implies the loss of Na and the formation of Na_2_Se_2_, which is the contiguous phase in the Na-Se phase diagram^61^. The pressure recorded for Na_2_Se_2_ is very small, but unlike selenium, Na_2_Se_2_ phase does not melt congruently, so a high pressure is not expected necessarily.

**Figure 12:**
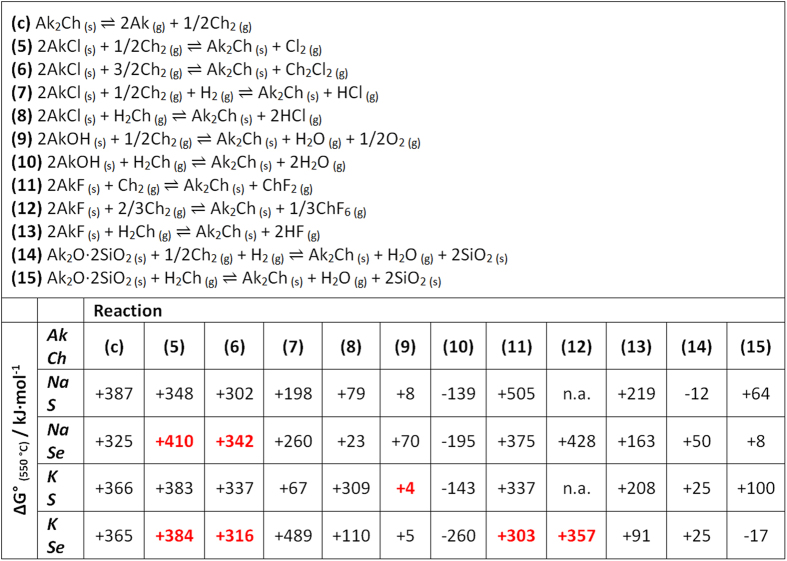
Values of standard Gibbs free energies expressed as kJ·mol^−1^ of Ak_2_Ch_(s)_, calculated at 550 °C for reactions (**c**) and (5–15), where Ak = alkali metal and Ch = chalcogen. The Gibbs free energy data is taken from^58^,^59^. The red highlighted data correspond to the reaction tested by TG-MS (9) and KEMS.

Atomic alkali species formed in (d) can either contribute to the Na doping by direct incorporation into the film via the gas-phase or react again with chalcogen in the gas-phase at another location within the chamber to re-form Ak_2_Ch. This last possibility is very favourable, seen the high Ak-Ch chemical affinities (reactions (c) in Fig. 12). The re-formed Ak_2_Ch could then re-deposit on all surfaces, including the film, leading to alkali doping. This classifies the combined (c) and (d) as chemical vapor transport reactions.

Route (e) consists in the formation of alkali polychalcogenides by reaction between Ak_2_Ch formed through (4) and the excess chalcogen supplied in the annealing chamber. The Na-S, Na-Se, K-S and K-Se equilibrium phase diagrams show that stable polychalcogenide phases form at Ch mole fractions higher than 0.5, and all these phases have far lower melting/peritectic temperatures than the refractory Ak_2_Ch^61^,^62^,^63^,^64^.

Hergert *et al*. proposed an interesting mechanism where sodium polyselenides, which form exothermically, exhibit a surface intermediary catalytic behavior during the CIGSe growth^65^,^66^ (like sodium polytellurides might be involved in the CdTe case^42^,^67^). This proposition was also adopted by Sutter-Fella *et al*. who described the sodium-assisted sintering of Cu_2_ZnSn(S,Se)_4_ as mediated by liquid Na_2_Se_x_ phases^20^. Although this mechanism seems to nicely explain the grain growth enhancement attributed to Na, as observed also here, the possibility of gas-phase transport via alkali polychalcogenides was not discussed.

**Figure 13:**
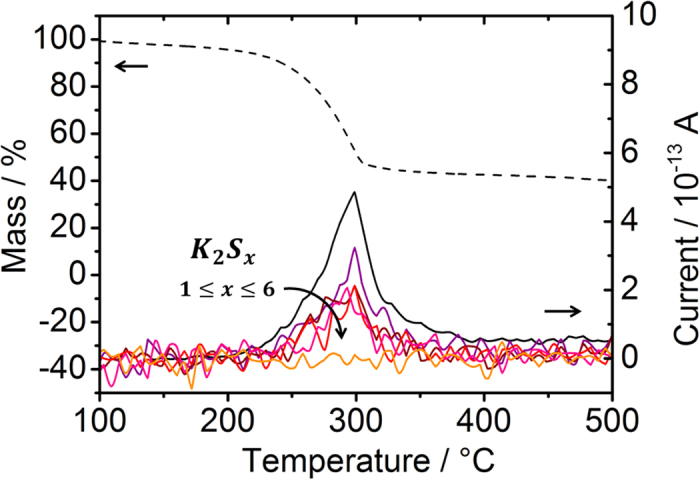
TG-MS analysis of a S:KOH 5:1 mole ratio mixture showing the evolution of K_2_S_x_ gas species presumably responsible for K gas-phase transport under the conditions investigated by Johnson *et al*.^1^. The black solid line corresponding to x = 1 has been divided by 10 in order to fit along with the x > 1 data. The dashed line corresponds to the thermogravimetric data.

Due to the lower density of charge, alkali polychalcogenides are naturally less ionic than the alkali chlorides, and are therefore expected to be more volatile. Once formed, it seems also possible that alkali polyselenides or polysulfides are conveyed into the gas phase by the flux of N_2_ or gaseous elemental chalcogen, even before an equilibrium vapor pressure can be established. Formation and gas-phase transport of alkali polychalcogenides could then contribute, via route (e), to the alkali transport observed here and in the previous works^1^,^25^,^26^. Evidence for this route is supplied in Fig. 13 for the case of the potassium-sulfur system.

The potential chemical reactions in Fig. 12 are discussed next, based on their calculated variations of Gibbs free energy. A range of different alkali sources are considered, such as chlorides, fluorides, hydroxides and silicates, with sulfur, selenium, hydrogen sulphide and hydrogen selenide.

All the Gibbs free energies of the potential reactions (5–15) are endoergonic (positive) except some involving silicates (14) and (15), taken here as a proxy for SLG, and reactions (10) that are acid-base reactions. The positive sign indicates that the reactions are not thermodynamically favourable at equilibrium. Nevertheless, an attentive observation of the physical state of the reaction products reveals that the formation of the alkali chalcogenide is associated with the release of gaseous species. Among the possible reactions suggested in Fig. 12, the high energy content of Cl_2_ makes reactions (5) particularly unfavorable compared to reactions (6) and (7), where more stable gas species such as the dichalcogen dichloride and hydrogen chloride are formed, respectively. These gas species have very high vapor pressures and are not likely to condense at the temperature and pressure employed in this work. As expected by Le Chatelier principle, the consequence of their removal from the reaction chamber is that the chemical equilibrium is shifted to the right, increasing the formation of alkali chalcogenide and making the reactions difficult to revert.

From this consideration it is expected that the type of reaction chamber – semi-open or closed – has a strong effect on the resulting extent of doping, with closed systems being likely to attain lower levels of incorporation. In this respect, reactions (9) and (10) involving the alkali hydroxides are among the most thermodynamically favorable, and are very likely to occur even in closed systems. For example, reaction (9) could well explain the results reported by Johnson *et al*., whose reaction chamber was a sealed quartz ampoule^1^.

The situation where sodium is released from SLG is described by reactions (14) and (15). Here SLG is substituted by the alkali disilicates where alkali are replaced by hydrogen atoms to desorb water molecules. Reactions (14) are only slightly unfavourable for Na/Se, K/S and K/Se, while for Na/S the reaction is actually exoergonic. This could explain the ease with which sodium *gas-phase* contamination occurs from routine use of SLG substrates. Likewise, reactions (15) are only slightly unfavourable for Na/Se and Na/S, while for K/Se the reaction is exoergonic.

The thermochemical data in Fig. 12 is also consistent with the differences observed in this work for the alkali incorporation via NaCl and KCl. It was shown that a similar increase of Na and Cl SIMS signals was attained upon NaCl doping, while with KCl the increase of Cl signal was higher compared to K. By comparison of reactions (6) and (7) in Fig. 12 for the Na and K cases, one can notice that the formation of Na_2_Se is less demanding than the formation of K_2_Se (+260 versus +316 kJ·mol^−1^). This means that before an appreciable amount of K_2_Se is able to form by reaction of KCl and Se, KCl vapor is able to reach the surface of the CIGSe film and get incorporated, simply via route (a). It can be argued that the Gibbs free energy difference is enough to ensure KCl incorporation despite KCl having a slightly lower vapor pressure than NaCl. Indeed, this happens because NaCl rather reacts with Se, perhaps even in the gas phase, leading to an increase of the activity of gaseous (poly)selenides at the expense of gaseous NaCl.

Reactions (11–13) correspond to the fluorides, the most commonly employed alkali salts for PDT. It is surprising to notice that they are not particularly favourable compared to the corresponding reactions of the alkali chlorides. Laemmle *et al*. have recently suggested the formation of SeF_6_ gas species to account for the limited amount of F detected in CIGSe films subject to NaF PDT compared to a reference sample not subject to PDT^49^. It was suggested that SeF_6_ molecules could form and desorb from the CIGSe surface when the evaporated NaF is exposed to Se, but no experimental evidence was shown. Actually, Fig. 12 shows that the formation of gaseous SeF_2_ (11) is thermodynamically more favourable than SeF_6_ (12). If the hydrogen chalcogenides are used instead of elemental chalcogens, the corresponding reactions (13) are more favourable, but still not spontaneous thermodynamically.

In order to test the hypothesis that alkali gas-phase transport can occur also via formation of alkali (poly)chalcogenide gas species, a preliminary KEMS experiment was performed on the Se:NaCl 5:1 mole ratio mixture employed for the annealing of sample *Q-Na-Gas*. Besides the selenium oligomers Se_n_^+^, with n = 2–7 and NaCl, no Na_2_Se_y_ molecule was observed up to the operating temperature used in the synthesis (around 550 °C). The (NaCl)_2_ dimer was also detected above 650 °C, but interestingly, the measured (NaCl)_2_/NaCl intensity ratio was about three orders of magnitude lower than expected for pure NaCl^68^ (5.7·10^−4^ instead of 0.39). This points to a low activity of NaCl in the evaporating solid, which is an indirect evidence for a reaction between Se and solid NaCl. Additional KEMS measurements were performed with a Se:KCl:KF 1:1:1 mole ratio mixture to test reactions (5), (6), (11) and (12). No appreciable signals corresponding to the most isotopically abundant masses of Cl_2_, Se_2_Cl_2_, SeF_2_ or SeF_6_, were recorded, which is consistent with the thermochemical computations in Fig. 12 (the minimum measureable pressure with the apparatus is 10^−8^ mbar). It should be noted that the close-to-equilibrium conditions of KEMS experiments are very different from the actual conditions used in selenization. Furthermore, KEMS does not allow to test reactions (7–8), (10), (13–15) involving gaseous hydrogen and hydrogen chalcogenides, because the measurements are performed in ultra high vacuum. For these reasons, further experiments were carried out with a different setup, a TG-MS instrument allowing to operate under flux. For this experiment a mixture of KOH and S was chosen. This is the mixture utilised by Johnson *et al*.^1^. It is among the most thermodynamically favourable reactions, as highlighted in red in Fig. 12, reaction (9). It has been chosen in order to increase the chances for any gas-phase K-S species to be detected. Given the different geometry of the TG-MS setup compared to KEMS, low volatile molecules are very likely to condense in the TG-MS instrument before attaining ionization and subsequent trapping by the quadrupole analyzer. For this reason, the special Skimmer interface has been employed (see experimental section). The results of the TG-MS analysis are shown in Fig. 13.

The TG-MS analysis confirms that species with mass consistent with potassium polysulfides are formed at temperatures compatible with the chalcogenization experiments performed here and in Johnson’s study. The solid residual of the TG-MS run is a complex mixture of reaction products, as revealed by XRD analysis (see supporting information). Nevertheless, species such as K_2_S (c*F*12-CaF_2_) and K_2_S_5_ (o*P*28-S_5_Tl_2_) appear to be formed, along with KHS (h*R*6-NiO). This happens despite the unfavourable thermochemistry of the reactions leading to the monochalcogenides formation (Fig. 12). It is rather surprising being able to detect K_2_S_x_ *gas phase* species at such low temperatures, given that previous reports indicate that K_2_S_(s)_ vaporizes incongruently producing predominantly potassium in the vapor phase at temperatures compatible with this work^69^, while much higher temperatures (>700 °C) are required to be able to detect K_2_S_(g)_^70^. However, these two works were performed under effusion conditions, i.e. close to equilibrium, starting from pure K_2_S_(s)_, so without excess of chalcogen. It is possible that gas-phase (poly)chalcogenide species might form under out of equilibrium conditions, such as those experienced by the rapidly evolving reactive mixtures employed here.

### Gas-phase alkali desorption

Given the solid-gas nature of the sodium and potassium incorporation investigated in the present work, it is legitimate to question whether such a process is ruled by an equilibrium chemical reaction or not^23^,^24^,^71^. That is to say: if absorption of alkali species at the surface of the chalcogenide film occurs from the gas-phase, can the same surface also incur desorption of such species? This would correspond to the reversed process, where the alkali species are released back into the gas-phase.

**Figure 14:**
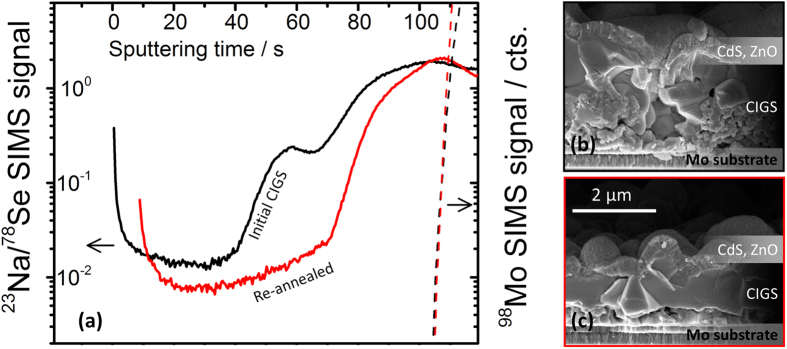
(**a**) ^23^Na/^78^Se SIMS depth profiles of a CIGS film obtained with intentional 0.6 at. % Na^28^ (black curve) and of part of the same film re-annealed under Se flux at 500 °C for seven minutes (red curve). The sputtering time scales are shifted linearly for comparison purposes (the ^98^Mo signals of the back contact are made to overlap). The corresponding SEM cross sectional images of the films are shown respectively in (**b**) and (**c**), as complete solar cell devices.

Figure 14a shows SIMS measurements of an annealed CIGS film where sodium was intentionally incorporated by dissolving NaCl (0.6 atomic %) in the precursor ink as described in Berner *et al*.^28^ (black line). Subsequently, the oven was cleaned and part of the same film was re-annealed in a flowing selenium flux at 500 °C for seven minutes (red curve). The corresponding cross sectional SEM images are also shown in Fig. 1b,c, respectively (as complete solar cell devices).

The SIMS compositional profiles reveal a net sodium loss from the CIGS film upon re-annealing under the Se flux. The cross sectional micrographs of the two films do show that the grain morphology is also changed. The film that has been re-annealed appears to be thinner, more compact and with larger grain size compared to the initial CIGS film. Since sodium tends to accumulate at the grain boundaries^49^, the decreased sodium content in the re-annealed film is also consistent with its larger grain size. It is proposed that the missing sodium has been lost via the gas-phase, according to one of the routes (a–e) proposed in section *gas-phase alkali generation and transport: how does it happen?* It appears logical to believe that sodium is actively stripped from the CIGS grain boundaries by the selenium flux, as per route (e).


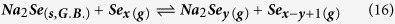


where 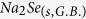 stands for the sodium (poly)selenide present in the solid state as adsorbed species at the CIGS grain boundaries^50^. Since the oven was cleaned and the sample was re-annealed in a flowing Se flux, the sodium vapour phase species is removed, forcing reaction (16) to the right.

Intentional depletion of sodium from polycrystalline CIGS films has been demonstrated by Forest *et al*.^72^ and Kitani *et al*.^73^ by cycles of deionized water rinse and air annealing of the films. Clearly, the liquid solution approach that they employed is different from the gas-phase study undertaken here. Nevertheless, the reversibility that they observed suggests that an analogous gas-phase process may also be reversible. This preliminary evidence is encouraging, but the ultimate confirmation of the phenomenon should be sought with a dedicated tool capable of providing a direct measurement of the evolved alkali-bearing gas species.

### Gas-phase alkali transport: discussion and implications

In principle, alkali gas-phase transport can occur through all routes (a-e) listed above. The contribution of each route must be evaluated on a case by case basis. Due to the lack of thermochemical data for the alkali polychalcogenide species, it is difficult to assign a precise mechanism of transport to back-up the Na experimental results shown in this work. Nevertheless, comparison with literature reports can help to contextualize the findings and provide useful insights.

If the transport of alkali species occurs by *in-situ* formation of alkali (poly)sulfide/selenides (b-e), the amount of alkali chalcogenide formed depends on the alkali source species employed be it halogenide, hydroxide or other and on the corresponding reaction thermodynamics (i.e. Fig. 12) and kinetics. If the transport occurs via congruent evaporation of the alkali source (a), the vapor pressure is dictated by the source itself. Therefore, in both cases, the level of doping may be tuned, to a certain extent, by carefully selecting the alkali source counterions.

The alkali source counterions and their by-products (e.g. reactions 5–15) may have secondary effects on the chalcogenide film growth, such as defect compensation, oxidation etc. Chemically speaking, if the route is via (b–e), any effect is expected to be minor when using the fluorides/chlorides, because the halogen-bearing products proposed for reactions (6–8) and (11–13) are relatively stable gas species. Detrimental effects seem certainly limited in the case of alkali fluorides, given that they are extensively employed in (solid state) post-deposition treatments attaining the highest efficient CIGSe devices^2^,^4^. This may also hold true for NaCl, since it was employed as a deliberate dopant leading to the highest reported efficiency for S-free CIGSe by solution processing (13.3%)^28^.

Compared to PDTs, alkali doping via the gas-phase through routes (b-e), appears to lead to different effects on the CIGSe surface, because the alkali counterion is either absent or composed of chalcogen. Indeed, in Reinhard *et al*.^43^,^44^ the presence of fluorides in direct contact with the CIGSe might play an important role on the surface nanostructuring. The cubic shape of the wells formed hints to a condensed state reaction between KF and CIGSe in the presence of Se. Therefore, the effect of gas-phase alkali incorporation into chalcogenide compounds is expected to confer properties that are different, in principle, to conventional PDTs. This aspect may be particularly relevant for applications where surface effects at interfaces play an important role, such as in CIGSe and other thin film PV.

To summarise, alkali incorporation can be achieved via the gas-phase (as shown here) or by evaporation/condensation followed by ion exchange with the already formed chalcogenide film (as in conventional PDTs).

Therefore, understanding the thermodynamic equilibria responsible for the gas-phase alkali transport (reactions (4) and (a–e)) is essential in view of (i) developing reliable gas-phase doping methodologies, and (ii) unravelling the chemistry of surface ion exchange in PDT^43^,^74^. Specifically, if vapor phase species can be absorbed and react with the CIGSe, they may also desorb and be lost from the CIGSe, as the preliminary evidence here suggests. This is the nature of gas-solid phase equilibrium reactions, and its consequences may become important in high vacuum synthesis procedures, e.g. physical vapor phase deposition.

More precisely, for case (i) depending on the ease to achieve a uniform gas phase distribution of the dopants, an effective dopant-chalcogenization treatment can be designed based on a static or on a forced convection flow system, as shown in this work. For case (ii), knowing the thermodynamics of the competing reactions occurring at the surface of the CIGSe during alkali fluoride PDT (i.e. also the gas-phase reactions) may help to better explain the phenomenon of surface nanostructuring^44^. Furthermore, it would be important in order to identify which alkali source best fits other chalcogenide systems being developed.

Future work should then focus on the synthesis and characterization of the single alkali polychalcogenides, with the aim of quantifying their vapor pressures, diffusion coefficients and Gibbs free energies of formation.

## Summary and conclusions

This work expands on previous reports of gas-phase alkali transport and incorporation in CI(G)Se^25^,^26^ and CZTS^1^, with the intent to clarify the origin of the transport.

Mixtures of alkali chlorides and elemental selenium are employed during reactive annealing of Cu-In-Ga metallic precursors under static and flux conditions. The gas-phase nature of the transport is supported by SIMS compositional data and is further confirmed by the spatially dependent optoelectronic properties of the resulting CIGSe-based devices produced from the flux annealing. Indeed, the sodium supplied via the gas-phase appears to improve the optoelectronic properties of CIGSe films in much the same way as other kinds of sodium incorporation routes. The known decrease of In-Ga interdiffusion upon Na incorporation is also observed and supported by SIMS, EQE and PL data. Contrarily, preliminary SIMS and PL evidence shows that KCl appears to promote In-Ga interdiffusion in CIGSe.

Based on mass spectrometric analyses and a critical assessment of calculated Gibbs free energies of reactions, the observed gas-phase transport is proposed to occur via five routes (a-e) and to originate from direct alkali source evaporation (a) and alkali monochalcogenide formation (4) (which is deemed possible thanks to the side-formation of uncodensable gas products). In principle, the alkali monochalcogenide formed can then evaporate (b), decompose and release neutral alkali atoms (c-d) or react with excess chalcogen to form more volatile polychalcogenide species (e).

KEMS analysis shows that Na_2_Se sublimation is unlikely to be the sole route for transport under equilibrium conditions. Static annealing shows that the transport with KCl+Se occurs differently from the NaCl+Se case. While limited amount of Cl is incorporated when using NaCl, both K and Cl levels increase substantially with KCl, suggesting that the transport of Na is also mediated by Se, while K is mainly transported via gaseous KCl. This is consistent with the thermochemical data corresponding to the formation of alkali selenides starting from the selenium-salts mixtures in H_2_/N_2_, which shows that Na_2_Se formation is more favoured compared to K_2_Se. As a result, KCl treatment leads to a CIGSe surface microstructure that is much smoother than that of the NaCl case. Such a microstructure is also very different from previously reported nanostructured patterns achieved by evaporation of KF followed by post deposition treatment^43^.

The absence of clear mass spectrometric signals for many of the species involved in the proposed reactions is consistent with the equilibrium thermodynamic calculations. Therefore, the gas-phase alkali transport observed here and implicitly acknowledged by most literature on NaF/KF post deposition treatments may imply that the proposed reactions deviate substantially from equilibrium under routine annealing conditions.

Preliminary evidence suggests that the gas-solid nature of the alkali metal incorporation in chalcogenide films is likely to be reversible, as shown by a decrease of the sodium SIMS signal in CIGS films re-annealed in selenium atmosphere.

### Outlook

From the proposed phenomenological analysis it is concluded that gas-phase alkali metal transport occurs through a plurality of routes and cannot be attributed to one single source. Its consequences lead to several considerations that go beyond the CIGSe and CZTS communities and encompass chalcogenide materials in general:

1. Background alkali contamination in standard synthesis equipments can be substantial and acts as a parallel doping mechanism alongside the standard doping routes. (Its effect on device optoelectronic properties can be substantial, and should be considered when doping mechanisms are studied in details.)
2. The alkali gas-phase reactions can be exploited for research on extrinsic doping, e.g. by formation of library samples with monotonic variation of the doping concentration^26^.
3. Alkali gas-phase reactions may alter the surface microstructure and composition of chalcogenide films, leading to modified interfaces in the final device. This may open a number of possibilities for surface/interface engineering. For example, the enhancing effect of potassium on In-Ga interdiffusion is very relevant for CIGS technology and should be studied more closely.
4. During conventional post-deposition treatments, gas-phase reactions appear to occur alongside condensed-state reactions with alkali fluorides at the surface of chalcogenide films.
5. Indirect evidence suggests that alkali metal incorporation in chalcogenide films is a reversible process. I.e. alkali species adsorbed from the gas-phase may also desorb the surface. Future works should aim at providing a direct proof of the phenomenon, as well as quantify and assess its consequences.

In all the scenarios above, a deeper knowledge of the chemical and physical properties of the individual alkali chalcogenides is necessary. This will be part of future research studies.

## Additional Information

**How to cite this article**: Colombara, D. *et al*. Deliberate and Accidental Gas-Phase Alkali Doping of Chalcogenide Semiconductors: Cu(In,Ga)Se_2_. *Sci. Rep.* **7**, 43266; doi: 10.1038/srep43266 (2017).

**Publisher's note:** Springer Nature remains neutral with regard to jurisdictional claims in published maps and institutional affiliations.

## Supplementary Material

Supplementary Information
