# Supplementary material for: Deliberate and Accidental Gas-Phase Alkali Doping of Chalcogenide Semiconductors: Cu(In,Ga)Se2
Source: Sci Rep. 2017 Feb 24;7:43266. doi: 10.1038/srep43266 (PMC5324121; doi:10.1038/srep43266)
Supplement: Supplementary Information [file srep43266-s1.pdf]

Supporting Information for: Deliberate and Accidental Gas-Phase Alkali Doping of Chalcogenide Semiconductors: $Cu(In,Ga)Se_2$

Diego Colombara[a], Ulrich Berner[b], Andrea Ciccioli[c], João C. Malaquias[d], Tobias Bertram[a], Alexandre Crossay[a], Michael Schöneich[e], Helene J. Meadows[a], David Regesch[a], Simona Delsante[f], Guido Gigli[c], Nathalie Valle[g], Jérome Guillot[g], Brahime El Adib[g], Patrick Grysan[g] and Phillip J. Dale[a]*

[a] University of Luxembourg, Physics and Materials Science Research Unit. 41, rue du Brill, L-4422 Belvaux, Luxembourg.
[b] Robert Bosch GmbH, Corporate Sector Research and Advance Engineering. Robert Bosch Campus 1, D-71272 Renningen, Germany.
[c] Università la Sapienza di Roma, Dipartimento di Chimica. Piazzale Aldo Moro 5 00185 Roma, Italy
[d] Department of Materials Engineering, KU Leuven, Kasteelpark Arenberg 44 – bus 2450, B-3001 Leuven, Belgium.
[e] NETZSCH-Gerätebau GmbH, Wittelsbacherstraße 42 D-95100 Selb/Bayern, Germany.
[f] Università degli Studi di Genova, Dipartimento di Chimica e Chimica Industriale, Via Dodecaneso 31 16146 Genova, Italy.
[g] Luxembourg Institute of Science and Technology. 41, rue du Brill, L-4422 Belvaux, Luxembourg.

**Corresponding author: Diego Colombara*
e-mail: diego.colombara@uni.lu
Telephone: 003524666446467

Solar cell parameters of CIGSe library samples

Table SI1 Solar cell parameters of CIGSe library samples selenized with the gas-flow apparatus

	Flux-clean				*Flux-NaCl*				*Flux-dirty*			
**#**	**Eff.**	**Voc**	**F.F.**	**Jsc**	**Eff.**	**Voc**	**F.F.**	**Jsc**	**Eff.**	**Voc**	**F.F.**	**Jsc**
**1**	2.56	252	36.8	27.6	7.89	402	56.8	34.5	6.85	363	53.7	35.1
**2**	2.16	246	31.8	27.7	6.96	411	50.4	33.7	7.30	365	56.2	35.6
**3**	1.95	264	31.1	23.8	7.48	405	52.8	35.0	7.72	373	56.1	36.9
**4**	1.51	262	30.7	18.7	6.06	382	43.9	36.1	7.43	368	54.2	37.2
**5**	1.58	263	31.6	19.1	4.88	352	38.9	35.6	7.06	369	51.1	37.5
**6**	1.93	274	33.9	20.7	4.57	345	37.6	35.2	6.76	361	50.1	37.4
**7**	2.56	286	37.2	24.1	3.88	317	36.1	33.9	6.98	362	51.3	37.5
**8**	3.39	301	41.8	26.9	4.15	352	35.7	33.0	6.82	362	51.0	37.0
**9**	3.94	311	43.5	29.1	3.17	295	35.4	30.4	6.95	356	53.2	36.7
**10**	4.08	316	42.2	30.6	3.62	307	38.9	30.3	6.47	350	52.0	35.6

Efficiency is expressed as %, Open circuit voltage (V_{OC}) is expressed in mV, short circuit current density (J_{SC}) is expressed as $mAcm^{-2}$ and fill factor (F.F.) is expressed as percentage.

Remarks on the significance of alkali contamination

As shown in Figure 5 of the main manuscript, the background ^{23}Na and ^{39}K signals of the blank CIGSe layer on quartz substrate (a) are not negligible, despite the sample was obtained in absence of intentional alkali sources. This aspect is important for the correct interpretation of a number of research studies on alkali metals doping, where lack of sound trends are lamented [1,2]. Especially for the case of sodium, its quasi ubiquitous presence can be the source of unexpected contamination [3], and therefore of confusion for the interpretation of experimental results. This was apparent already in Wieting's and Johnson's works [4,5] where unintentional sodium doping of $Cu(In,Ga)Se_2$ and Cu_2ZnSnS_4 were achieved simply by loading soda-lime glass (SLG) in the reaction chamber employed for the chalcogenization of the metal precursor films.

In this work it was found that meaningful sodium incorporation in $Cu(In,Ga)Se_2$ can be achieved even if no SLG is introduced during the selenization, but simply if the process is run in sequence after a run where SLG substrates were used (**Figure SI1**).

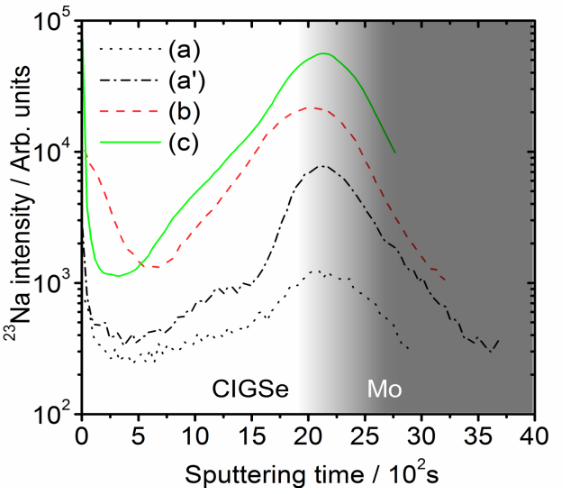

Figure SI1 Semilogarithmic plots showing ^{23}Na compositional profiles of samples Q-Na-Blank (a) and of the same precursor selenized after a run where SLG substrates were employed (a'). The profiles of sample Q-Na-Gas (b) and SLG-K-Blank (c) are also included for comparison from Figure 5. The shaded area is a guide for the CIGSe/Mo interface location.

Table SI2 Series of annealing experiments performed to assess sodium contamination in annealing apparatus.

**#**	**Sample**	**Description**
1	Blank	As deposited Mo-coated quartz substrate
2	Clean oven	Mo-coated quartz substrate annealed at 550°C in graphite box and new quartz tube cleaned at 800°C under N2 flux for 24 h
3	Clean oven + Se	Mo-coated quartz substrate annealed at 550°C in the presence of 100 mg Se
4	CIG 1	CIG precursors annealed at 550°C in the presence of 100 mg Se
5	CIG 2	CIG precursors annealed at 550°C in the presence of 100 mg Se and NaCl
6	Dirty oven + Se	Mo-coated quartz substrate annealed at 550°C in the presence of 100 mg Se without NaCl
7	Dirty oven	Mo-coated quartz substrate annealed at 550°C with no selenium and no NaCl

A series of annealing experiments has been performed, as described in Table SI2, with the aim of assessing the sodium contamination of the annealing apparatus before and after the introduction of intentional sodium sources and its persistence in the absence of sodium sources. Fig. SI2 shows the SIMS compositional depth profiles of ^{23}Na and ^{16}O.

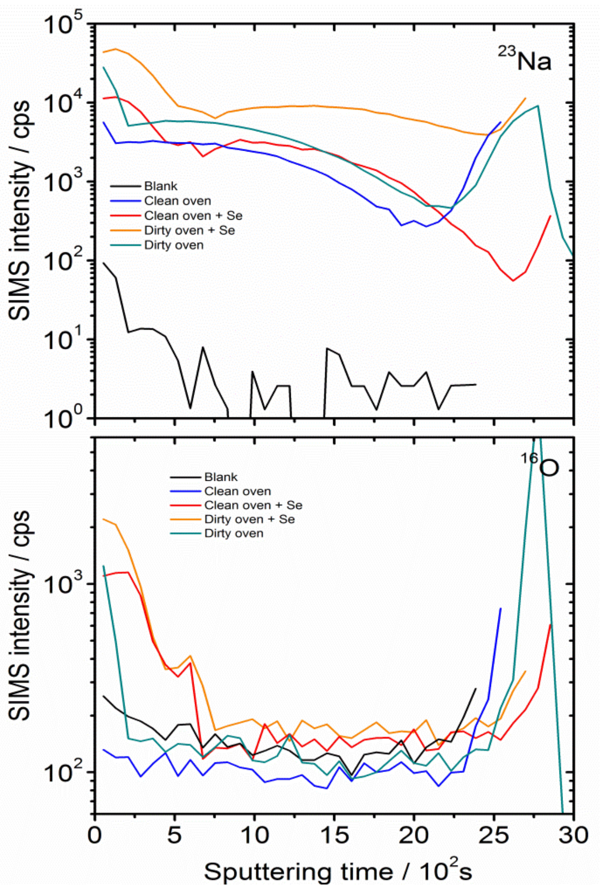

Figure SI2 Semilogarithmic plots showing ^{23}Na and ^{16}O compositional profiles of the series of samples described in Table SI2, aimed at assessing intentional sodium contamination persistence in the selenization apparatus.

The background sodium concentration of the blank molybdenum coated quartz slide is very low, as revealed by the solid black line in Fig. SI2 (run # 1 in Table SI2). The SIMS signal increases by approximately 5 orders of magnitude as soon as the blank Mo is annealed without selenium at 550 °C in the furnace that has been carefully cleaned with deionized water and by flushing nitrogen at 800 °C for 24 hours (blue line, run # 2). The level of sodium increases further but not dramatically, upon annealing at 550 °C in the presence of Se (red line, run # 3). At this point the furnace was loaded with a Cu-In-Ga precursor film and a selenization was run in the absence of intentional sodium species (run # 4), followed by a similar annealing in the presence of Se and intentional NaCl as sodium source (run # 5). The background level of sodium in the furnace was then re-assessed in a subsequent test on a Mo blank annealed in the same furnace at 550 °C in the presence of just Se (yellow line, run # 6). The increase of sodium concentration in the film is apparent compared to the film annealed in the furnace before the sodium source was ever introduced. This suggests that the sodium species formed during the annealing permeate the annealing apparatus and are then re-dispersed in subsequent annealings, even in the absence of intentional sodium sources. The last test (green line, run # 6) corresponds to the final contamination level in the furnace assessed by measuring the sodium SIMS signal of a blank Mo annealed in at 550 °C in the absence of Se. It appears that the sodium contamination is decreased compared to run # 6, but still higher than the background level present during run # 3. A correlation was also observed between the compositional profile of Na and O, but its origin was not investigated further.

XRD analysis of the solid residual of TG-MS KEMS runs

The residual of the TG-MS and KEMS runs appeared to be hygroscopic. The obtained XRD patterns are shown in **Fig. SI3** and **SI4**. The high background is mainly due to the low crystalline quality of the specimens. In addition, the preparation of the sample induced a preferential orientation for some phases (e.g. for the K_2S_5 compound). The mixture appears to be quite complex, as several phases have been identified: three different sulphur allotropic forms, sulphides such as K_2S (c*F*12-CaF_2) and K_2S_5 (o*P*28-S_5Tl_2), two different polymorphic forms of K_2SO_4, $K_2S_3O_6$ (o*P*44-$K_2S_3O_6$) and KHS (h*R*6-NiO).

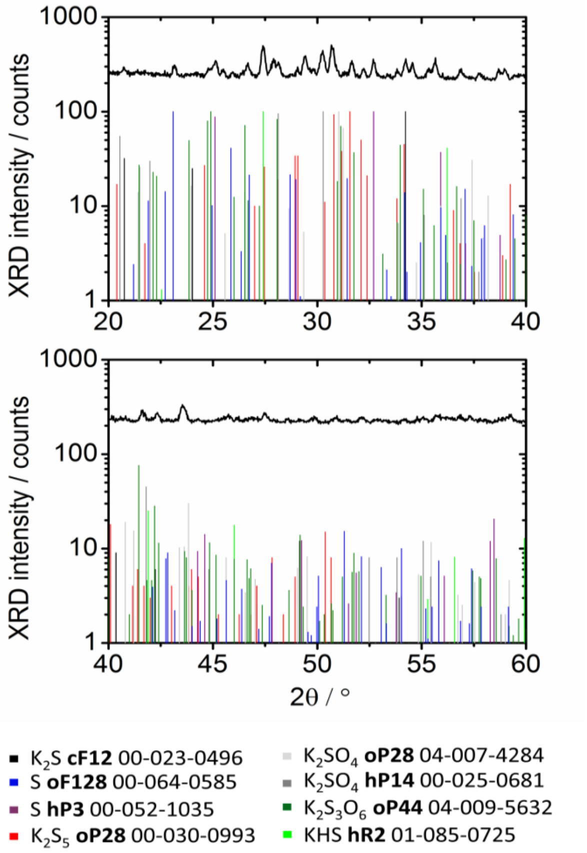

Figure SI3 XRD pattern of the residual of the mixture of KOH and elemental sulfur after the thermal treatment performed with the TG-MS instrument. The reference patterns of the phases that are most likely present are also shown (see legend).

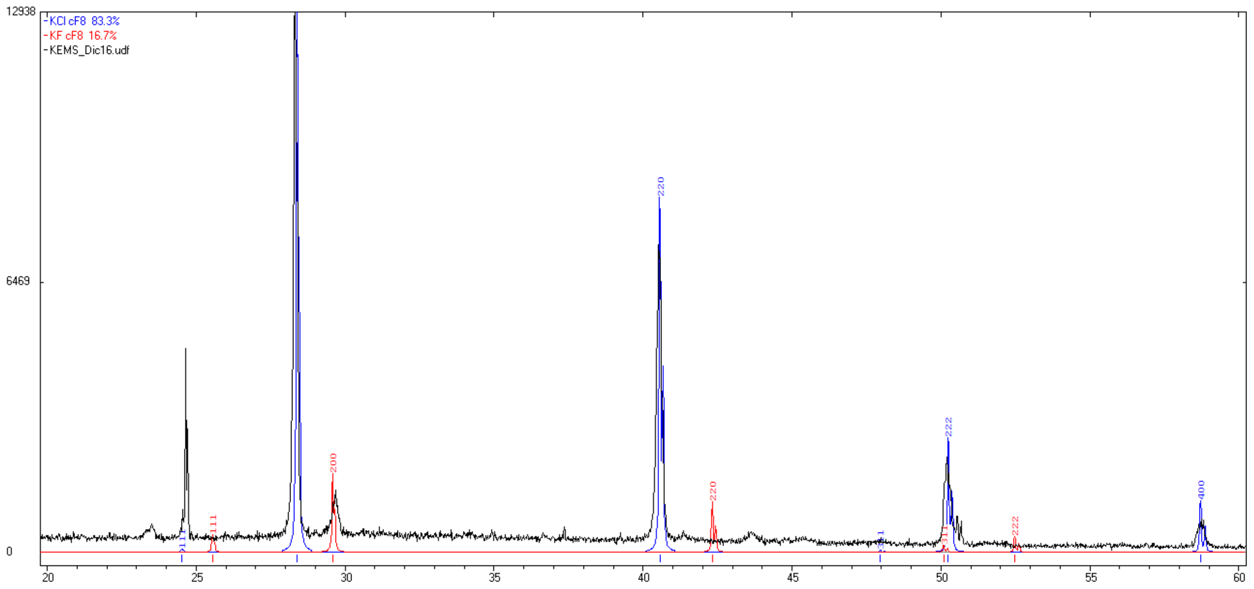

Figure SI4 XRD pattern of the residual of the mixture of KCl+KF+Se after the thermal treatment performed with the KEMS instrument. The reference patterns of KCl and KF are shown, but only KCl is identified.

References

1. Rockett, A. The effect of Na in polycrystalline and epitaxial single-crystal CuIn1−xGaxSe2. *Thin Solid Films* **480–481,** 2–7 (2005).
2. Rockett, A. *et al.* Na in selenized Cu(In,Ga)Se2 on Na-containing and Na-free glasses: distribution, grain structure, and device performances. *Thin Solid Films* **372,** 212–217 (2000).
3. Constant, I., Tardif, F. & Derrien, J. Deposition and removal of sodium contamination on silicon wafers. *Semiconductor Science and Technology* **15,** 61 (2000).
4. Wieting, R. *et al.* CIS thin film manufacturing at Shell Solar: practical techniques in volume manufacturing. in *Conference Record of the Thirty-first IEEE Photovoltaic Specialists Conference, 2005* 177–182 (2005). doi:10.1109/PVSC.2005.1488100
5. Johnson, M. *et al.* Alkali-metal-enhanced grain growth in Cu2ZnSnS4 thin films. *Energy & Environmental Science* **7,** 1931–1938 (2014).
